# Abortion practices among women in Buea: a socio-legal investigation

**DOI:** 10.11604/pamj.2019.32.146.17732

**Published:** 2019-03-26

**Authors:** Mbuwir Charlotte Bongfen, Essimien Elizabeth Bessem Abanem

**Affiliations:** 1 Department of Population, Family and Reproductive Health, School of Public Health, University of Ghana, Ghana; 2 Department of Sociology and Anthropology, Faculty of Social and Management Sciences, University of Buea, Buea, Cameroon

**Keywords:** Abortion, laws, knowledge, determinants, Cameroon

## Abstract

**Introduction:**

there are controversies surrounding the practice of abortion especially in developing countries of Africa. Cameroon is not an exception to this and hence this study aims at assessing knowledge on the awareness of abortion laws, the factors that determine abortion and people’s perceptions on the legality of abortion in Cameroon.

**Methods:**

the study is cross-sectional in its design. A total of 224 women were randomly sampled. Data for the study were collected through the use of questionnaires from the sampled women of child bearing age. These were used to assess knowledge on the awareness of abortion laws and the determinants of abortion. The data were analysed using STATA 15.

**Results:**

the prevalence of induced abortion was 21%. The major determinants of abortion among these women were; desire to stay in school (28%), fear of parents (24%) and shame of being pregnant out of wedlock (26%). Furthermore, many women are not aware of the situations where abortion is allowed and hence some still undertake illegal abortions even when they find themselves in situations deserving a legal abortion.

**Conclusion:**

induced abortion is still common in Buea, Cameroon despite the fact that it is illegal. Cameroon’s legal and health system needs to work in harmony in order to lessen the legal processes of having a legal abortion.

## Introduction

There are controversies surrounding the practice of abortion especially in developing countries of Africa. While a number of people in these countries, given their religious and cultural backgrounds, frown at this practice, the growing unsafe cases of abortion is making development stakeholders of some of these countries to consider legislating otherwise on the act. The World Health Organization defines an unsafe abortion as a procedure for terminating an unwanted pregnancy either by persons lacking the necessary skills or in an environment lacking the minimal medical standards or both [1]. Abortion is an age old practice carried out by human beings. It is practiced in all parts of the world though still widely illegal. Unsafe abortion is one of the neglected problems of health care in developing countries [2]. In fact, in most places, it is shrouded in secrecy, which makes it difficult to determine the exact incidence of the condition. Recent estimates gives an overall figure of around 30 million induced abortions annually in the world [3]. About 19-20 million of these abortions are done by individuals without the requisite skills or in environments below minimum medical standards or both (ibid). Nearly all unsafe abortions (97%) are in developing countries [4, 5]. In Africa, 4.2 million abortions are estimated to take place per year, with an unsafe abortion rate of 22 per 1000 women, or one unsafe abortion per seven live births [6, 7].

In Cameroon, the prevalence of induced abortion is about 25% [8]. Furthermore, in 2000, the estimated maternal mortality ratio attributed to unsafe abortion in Cameroon was 90 to 100,000 live births [6]. This is a huge number which could be controlled if abortions were done safely. Unsafe abortion is responsible for 25% of maternal deaths in Cameroon [8]; consequently, abortion is one of the leading causes of maternal mortality in Cameroon. As a result of these maternal deaths arising from abortion, there are ongoing debates on the liberalization of abortion in Africa [9] generally and Cameroon in particular. There is thus the need to reverse the increasing trend of maternal mortality in Cameroon given the significant contribution of women to family welfare and to the development of society. The law in Cameroon under the 2007 Penal Code (sections 337-339) stipulates that the performance of abortions is illegal except if proven necessary to save the mother from grave danger to her health or when the pregnancy is the result of rape. It continues that anyone performing an illegal abortion is subject to one to five years imprisonment and a fine of 100,000 to two million CFA francs. A woman who procures or consents to her own abortion is subject to imprisonment for fifteen days to one year and/or a fine of 5,000 to 200,000 CFA francs. Despite these harsh legal sanctions meted on those involved in the act such as 10 years of imprisonment and withdrawal of the certificate of medical practitioners, unsafe abortion is still on the rise. Clandestine abortion services are offered by lay abortionists, trained midwives and native doctors, out of view of the public health services which result in many cases of poorly performed abortions [10].

The Cameroon penal code Chapter V. Children and the family Section 337 states as follows: (1) Any woman procuring or consenting to her own abortion shall be punished with imprisonment from fifteen days to one year or with fine from five thousand to two hundred thousand francs or with both such imprisonment and fine. (2) Whoever procures the abortion of a woman, notwithstanding her consent, shall be punished with imprisonment from one to five years and with fine from one hundred thousand to two million francs. (3) The penalties prescribed by subsection (2) shall be doubled where the offender: (a) engages habitually in abortion; or (b) practices the profession of medicine or an allied profession. (4) In the circumstances of subsection (3) (b), the court may also order closure of the professional premises and impose a ban on his occupation under sections 34 and 36 of this code. This abortion laws are highly restrictive and have been there for a long period of time. The penal code was revised in 2007 and since then the laws have remained the same. Based on these laws, women are not allowed to seek abortion on demand. This has, however, not stopped abortions as the rate of abortion as unsafe abortions are widespread and are mostly conducted illegally [11]. Therefore, many of the perpetrators of illegal abortion go unpunished as the women who are their clients will not reveal their identities except in cases where the abortions end up with further complications.

The law, however, allows abortion to safe the woman’s life, preserve physical and mental health and in the case of rape or incest. This law is still misconstrued because the law further denotes that: *“The doctor shall obtain the opinion of two experts each chosen respectively from legal experts and members of the National Council of Medical Practitioners. The latter shall testify in writing that the life of the mother can only be safeguarded by means of the intervention. The protocol of consultation shall be made in 3 copies one of which shall be handed to the patient and the other two to the consultant physician and legal expert. Besides, a protocol of the decision taken shall be sent by registered mail to the chairperson of the National Council of Medical Practitioners.”*

With this addition to the law, it makes it difficult obtaining a legal abortion even when a woman deserves it. For instance, a woman who has been raped in a rural area might not even have access to two doctors from the said national council. This further makes it more complicated even in situations of legal abortions. In urban areas where there are doctors who can give their opinion, the whole legal process becomes lengthy and the final decision to have a legal abortion could take long.

**Research objectives:** the goal of this study is to examine the abortion practices among women visiting health facilities within the Buea Health District in Cameroon. Specifically, the study: assesses knowledge and awareness of abortion laws; evaluates the determinants of abortion among women in Buea, Cameroon; examines the barriers to effective legalisation of abortion in Cameroon.

## Methods

**Study design:** this study is a cross-sectional study. Women of child bearing age were recruited in some selected health facilities in Buea.

**Data collection:** this study used a questionnaire to collect the required data. The questionnaire was interviewer-administered and recruited women of child bearing age between the period June and July, 2018. The sample size for the study was estimated using the Cochrane’s formula for cross-sectional studies. The calculated sample size was 187 and after adjusting for a non-response rate of 20%, the total sample size was 224. A proportion of 26% for proportion of induced abortion from Ngowa *et al.* (2015) [12] was used to calculate the required sample size. These women were informed about the study and those who voluntarily consented were included in it. The study excluded women who were less than 18 years at the time of the survey as they could not consent. In total, data were collected from 224 women. Data were collected from women in four randomly selected health facilities. The women who accepted to participate were administered questionnaires as they were waiting to consult or for laboratory results. Women who were care givers or who came to visit patients were also invited to participate. Those who were critically ill were excluded from the study. The questionnaire was developed based on the objectives of the study and had both open-ended and close-ended questions. Participants answered questions on their demographic factors, clinical history of pregnancies and abortions, reasons of abortion, abortion laws, perceptions on the laws and suggestions on the way forward. Before commencing the study, the questionnaire were pretested in one of the health facilities that was not part of the study and some questions were rephrased to ease understanding before the data collection process. Data obtained from the questionnaires were used to assess the determinants of abortion as well as knowledge and awareness of abortion laws in Cameroon.

### Data analysis

The collected data were quantitative and were entered into excel on a daily basis throughout the data collection period. Questionnaires were doubled checked before being keyed into excel. The data were then analysed using STATA 15. Demographic characteristics were summarized with frequencies and percentages as shown on Table 1. The prevalence of induced abortions was also reported and comparisons were made between study participants using their demographic characteristics as independent variables and the induced abortion as the dependent variable. Bivariate analysis techniques were employed in this case using the t-test statistic to make inferences. A 95% level of significance was considered with a p-value of less than or equal to 0.05 used in judging the statistical significance of the results.

**Table 1:** demographic characteristics of participants

Occupation	Study population	Women who had an induced abortion	Women who have not had an abortion	P-Value
Student	64 (31)	15	49	-
Business	47 (23)	10	37	0.68
Nurse	23 (11)	6	17	0.92
Hairdresser/seamstress	44 (21)	8	36	0.54
Others	29 (14)	7	22	0.65
**Educational level**				
No formal education	10 (5)	2	8	-
Primary	10 (5)	7	3	0.67
Secondary	110 (55)	22	88	0.99
Tertiary	70 (35)	15	55	0.23
**Religion**				
Muslim	8 (4)	2	6	-
Christian	187 (96)	44	143	0.73

### Ethical issues

Ethical approval to conduct this study was obtained from the Faculty of health Science Institutional review board of the University of Buea, Cameroon. Administrative approval was then obtained from the South West Regional Delegation of health, Buea. Participation in the study was voluntary and participants signed a written consent form if they agreed to participate. Data collected for the study was kept confidential by the principal investigator and has been used strictly for the purpose of this study.

## Results

The study enrolled 224 women attending selected health facilities in Buea between June and July of 2018. The mean age of these women was 22.3 years with a standard deviation of 2.46. Most of the women were Christians (96%) of various denominations and among the four major occupations considered in the study, the majority (31%) were students. The reason for the dominance of Christians was because the Christian religion dominates in this part of Cameroon owing to the works of the early Christian missionaries who established their base at the coast of Cameroon in the 18^th^ Century. The dominance of students is further justified by the fact that Buea is largely an educational town with institutions of secondary and higher learning attracting students from other parts of Cameroon especially from within the English Speaking part. A summary of the demographic characteristics is presented in Table 1.

### Abortion practices and determinants

Among the women who participated in the study, 46 had undertaken an induced abortion at least once. This gave a proportion of 21%, implying that, one out of every 15 women had carried out an abortion. These abortions were shown to have some predisposing factors amongst which were; avoiding humiliation or ridicule from people around, fear of parents, not ready for children, not married, the desire to stay in school and in situations where the man responsible for the pregnancy did not accept it. Table 2 summarizes the responses of the participants. Among the predisposing factors of abortion, the desire to stay in school by the respondents is the highest determinant. This is justified by the fact that most of the participants indicated to be students and fell within the mean study age of 22.3 years.

**Table 2:** determinants of abortion

Reasons for Abortion	Proportion (%)
Desire to stay in school	28
Fear of parents	24
Shame/Humiliation	26
Has a child or children already	4
The man denies responsibility	6
Financial difficulties	10
Want to travel out	2

### Participants’ perception of the safety and legality of abortion practice

One of the study objectives was to understand participants’ perception of safe and unsafe abortions as well as their views on legalizing abortion. Out of the 224 women who participated in the study, only 40(20%) knew what a safe abortion was. The others had varied understanding of a safe abortion; some were of the view that, a safe abortion was one carried out when the pregnancy was less than two months while others thought a safe abortion was performed in a hospital. Additionally, the perceptions of the participants on effective legalization of abortion were also investigated. From the 220 participants who responded, 23% preferred that abortion should be legalized. Among the participants who agreed on the need for the legalization of abortion, 63% of them had carried out an induced abortion at least once. Most of the study participants were against legalizing abortion even though some had been involved in it. One of the reason for refusing this legalisation was that, it was going to allow women the liberty to indulge into unsafe sexual activities and increase not just the incidence of abortions but expose girls and women to other consequences of unprotected sex like sexually transmitted infections. Some women even indicated that the future consequences of abortion were worse than the abortion itself. Furthermore, given that most of the women were largely Christians, it was expected that they will be against the legalization of abortion on moral basis. The results also indicated that only few women were aware of their legal right to abortion in the case of rape or incest (37%). Moreover, only 5% of this number understood the whole process of obtaining a legal abortion. As for post-abortion complications, 25% of the respondents had experienced continuous bleeding and infections. The greater proportion of the abortions ended up without problems.

### Financial costs involved in abortion practices

The study went further to assess the cost involved in abortions. The average cost of an abortion incurred by participants was XAF 29,000 ($48). The minimum cost of an abortion was XAF 20,000 ($33) and the maximum was XAF 40,000 ($67). Abortions carried out in the hospitals were more expensive than those out of hospital. This could be one of the possible explanations as to why some women still prefer out of hospital abortions. It is on this basis that the study noted that safe and unsafe abortion practices are both carried out in the area of study. This could, partly, justify the reason why 25% of them reported to have had post-abortion complications.

## Discussion

The findings reported induced abortion at 21% and this was lower than that of Ngowa *et al.,* (2015) [12] that reported a proportion of 26% for women who had at least one induced abortion. From the 21% prevalence reported, 11 (23%) had undertaken more than one induced abortion. This also confirms the results of Feldman (2017) [13] that reported induced abortion as a common practice in Cameroon. Some of the abortions were done in medicine stores and at home (23%) on grounds that they were cheaper. This also ties with the findings of Bain and Kongnyuy (2018) [11] which reported that many abortions, including those provided for by the law, were done without following the established legal procedures. For the determinants of abortion, the desire to stay in school was the most reported reason for abortion and this came from students. This was possible as students believe that carrying a pregnancy to term while in school comes with a lot of sacrifices. However, it is important to note that higher institutions of learning in Buea in particular and Cameroon at large do not penalize students for getting pregnant. Indulging in abortion because of shame was also reported by 26% of the participants. This is probably due to the traditional social construct where women who get pregnant before marriage are seen as poorly behaved even when they are above age 18 years. It is seen as having mistimed motherhood which is unacceptable within most communities in Buéa and Cameroon generally. These findings also corroborate with those of Johnson-Hanks [14] in a study in Cameroon that equally reported fear of being humiliated as one of the pre-disposing factors of abortion. Financial difficulties, wanting to travel out of the country as well as situations where the supposed father of the child refused the pregnancy were also identified as some reasons why women engage in abortions.

Furthermore, some women reported cases of complications from abortions, this might have been due to poorly conducted illegal abortions. Some of the participants indicated to have even had the abortions at home (by using some herbal concoctions) or in some pharmacies in the quarters. The justification for this was the financial demands in modern health facilities which they cannot afford (given an average abortion cost of XAF 29,000 or $48) as well as making it discrete from people. This goes to confirm the high incidence of illegal abortions that go on unnoticed within the communities. As per the cost of abortions, the cheaper abortions were carried out in medicine stores clandestinely in the community while the expensive ones were done in hospitals and by medical doctors. Women who had carried out abortions out of hospital explained that it was relatively cheaper and more confidential than when doing so in the hospital. From the perceptions of the participants, legalizing abortion might not necessarily guarantee safety as women might still prefer abortions out of hospitals. In addition, legalizing abortion may not necessarily increase the number of women who will seek induced abortions as they still consider moral laws of Christianity on abortion. Moreover, in the developed world where the laws are less restrictive, the occurrence of abortion has not necessarily increased [15]. However, considering the Cameroonian content, it might be challenging to have it effectively legalized. This might be partly due to the influence of religion that forbids abortion in all its terms given that much of Cameroon is dominantly Christian. Furthermore, culturally, in the moral context of Cameroon, abortion is also unacceptable and hence having it legalized might still be far ahead in the future. Furthermore, legality it might not necessarily guarantee access [15] especially if the process of obtaining a legal abortion remains complex.

## Conclusion

Induced abortion is still common in this part of Cameroon despite the fact that it is illegal except under incest or rape. The law accepts abortion under certain circumstances like rape, incest or when the mother’s life is threatened due to the pregnancy. Despite this legal provision, women faced with such situations still have abortions without following the prescribed legal procedures due to their ignorance of the law and/or lengthy due processes of obtaining a legal abortion. The majority of women prefer the abortion law stays restricted based on religious ground. However, most women who have undertaken an abortion prefer it were legalized. The Cameroon legal and health system needs to work in harmony and maybe revisit the law so as to lessen the legal processes of having a legal abortion. While women wait for a review or rejuvenation of the law, it is necessary to engage and involve them more in safe and convenient family planning practices that can prevent unwanted pregnancies in the first place. Furthermore, there is the need to create awareness on the existing laws and how a women who is a victim of rape or incest could get access to a safe and legal abortion.

### What is known about this topic

- Studies have previously been done to determine the prevalence of unsafe abortions, reasons and consequences in Cameroon;
- However, there are limited studies assessing knowledge of abortion laws and determinants of abortion in Cameroon

### What this study adds

- This study intends to discuss knowledge on the awareness of abortion laws in Cameroon, the factors that determine abortion and people’s perceptions on the legality of abortion in Cameroon.

## Competing interests

The authors declare no competing interests.
